# Difficult medical encounters and job satisfaction - results of a cross sectional study with general practitioners in Germany

**DOI:** 10.1186/s12875-018-0747-0

**Published:** 2018-05-09

**Authors:** Katja Goetz, Janis Mahnkopf, Anna Kornitzky, Jost Steinhäuser

**Affiliations:** Institute of Family Medicine, University Hospital Schleswig-Holstein, Campus Luebeck, Ratzeburger Allee 160, 23538 Luebeck, Germany

**Keywords:** Cross-sectional study, Difficult encounter, General practitioner, Job satisfaction, Primary health care, Psychiatric condition

## Abstract

**Background:**

In primary care 15% of patient encounters are perceived as challenging by general practitioners (GP). However it is unknown what impact these encounters have regarding job satisfaction. The aim of this study was to evaluate which encounters are perceived as challenging by German GPs and whether they were associated with job satisfaction.

**Methods:**

A total of 1538 questionnaires were sent to GPs in the federal state of Schleswig-Holstein, Germany. GPs should rate 14 medical conditions and 8 traits of patients on the perceived challenge using a Likert scale (1: ‘not challenging at all’ to 10: ‘extremely challenging’). Job satisfaction was measured with the Warr–Cook–Wall job satisfaction scale. A linear regression analyses were used to explore potential associations between for the primary outcome variable ‘overall job satisfaction’.

**Results:**

Total response was 578 (38%). GPs perceived 16% of their patients as challenging. Psychiatric disorders such as somatization disorder (mean = 7.42), schizophrenia (mean = 6.83) and anxiety disorder (mean = 6.57) were ranked as high challenging while diabetes mellitus type 2 (mean = 4.87) and high blood pressure (mean = 3.22) were ranked as a rather low challenging condition. GPs were mostly satisfied with ‘colleagues’ (mean = 5.80) and mostly dissatisfied with their ‘hours of work’ (mean = 4.20). The linear regression analysis showed no association with challenging medical conditions and traits of patients but only with different aspects of job satisfaction concerning the outcome variable ‘overall job satisfaction’.

**Conclusions:**

Especially psychiatric conditions are perceived as challenging the question arises, in what amount psychiatric competences are gained during the postgraduate specialty training in general practice and if GPs with a mandatory rotation in psychiatry perceive these conditions as less challenging. Interestingly this study indicates that challenging encounter in terms of challenging medical conditions and traits of patients do not affect GP’s job satisfaction.

**Electronic supplementary material:**

The online version of this article (10.1186/s12875-018-0747-0) contains supplementary material, which is available to authorized users.

## Background

There is some evidence, that General Practitioners (GP) might perceive 15% of their patients as “difficult” which mostly include psychiatric disorders such as somatoform disorder, panic disorder, generalized anxiety and depressive disorder [1, 2]. Common medical disorders such as hypertension, diabetes, cardiac disease, or cancer were not perceived as difficult from the perspective of GPs [1, 2]. The occurring difficulties are based on factors that are due >to the patient, the physician, the situation or a combination of these factors [3, 4]. Because of this multi-directional relationship, the term ‘difficult encounter’ rather than ‘difficult patient’ is suggested [5]. Difficult encounters are capable of frustrating the patient and physician, for which both are responsible [3, 4]. When focusing on the patient’s factors, besides their medical condition, their personality and way of dealing with their condition can be a challenge for the GP [6]. GPs difficulties dealing with challenging encounters might also be based on a lack of empathy and understanding, when it comes to doctor-patient communication [3]. Therefore, communication models to improve communication skills for challenging encounters were developed [3, 4]. Challenging encounters can also be caused by missing expertise concerning a specific medical condition and the feeling to be compromised as a healer in such a situation [3].

GPs with a great amount of challenging encounters report lower job satisfaction [7]. Reasons for this are multiple. For example, challenging patients are often described as ‘high utilizers’ of the health care system and, by that increase workload for the GP [4]. High workload affects job satisfaction [8]. This endanger physician’s health: Physicians with high workload and low job satisfaction are more likely to go on to experience substance abuse, burnout, depression or death [7, 9]. Another finding is that low job satisfaction and low physician’s wellness lead to poorer quality of patient care [9]. Thus job satisfaction could be an important factor and acts as an indicator for the quality of care.

The aim of this study was to evaluate which encounters are perceived as challenging by German GPs and whether they were associated with job satisfaction.

## Methods

### Design and participants

The study was confirmed to the STROBE-Guidelines (Strengthening the Reporting of Observational Studies in Epidemiology) [10]. This cross sectional study was conducted in Schleswig-Holstein, a federal state in northern Germany. A total of 1538 questionnaires were sent to all GPs of this federal state by mail. In Germany the expression GP includes general internists and general practitioners. Therefore, there was no differentiation between these groups in the questionnaire. As an alternative for completing the questionnaire there was a short-response-sheet which could be completed and returned instead. The addresses of the GPs’ surgeries were obtained from the website ‘https://arztsuche.kvsh.de/’ of the regional Association of Statutory Health Insurance Physicians. No reminder was sent out. The survey was conducted between June and July 2015. The return of the anonymous paper-based questionnaire was classified as informed consent. Because this was an exploratory study, no power calculation was determined.

### Measures

Personal and practice characteristics were measured in the questionnaire including gender, age, duration of employment in the practice and the location of the practice (measured by the car registration number of their administrative district). The duration of employment as GP was grouped in four groups: ‘less than 5 years’, ‘5 to 10 years’, ‘11 to 20 years’ or ‘more than 20 years’. Moreover, the location of the practice measured by the car registration number was grouped into urban, medium-size town and rural area on the basis of the Federal Institute for Research on Building, Urban Affairs and Spatial Development (BBSR) [11].

Job satisfaction was measured with the German modified version of the Warr-Cook-Wall job satisfaction scale developed by Warr et al. [12, 13]. It is a well-known instrument which was validated in a large cohort of Australian medical practitioners [13]. This instrument consists of 10 items, overall job satisfaction (1 item) and 9 items to different aspects of satisfaction with work (amount of variety in job, opportunity to use abilities, freedom of working method, amount of responsibility, physical working condition, hours of work, income, recognition for work, and colleagues and fellow workers). Each item is rated on a 7-point Likert scale (1 = extreme dissatisfied to 7 = extreme satisfied). A higher overall mean score indicates higher job satisfaction. Cronbach’s α of the job satisfaction scale in this study was 0.607.

GPs were asked to estimate the percentage of challenging patients in their practice. Difficult medical encounters were operationalized with respect to two subjects: challenging medical conditions and challenging traits of patients. For the measurement of how challenging GPs perceive different medical conditions and different traits of patients, a questionnaire was developed consisting of 14 medical conditions and 8 traits. The conditions and traits chosen were defined by a selective literature search [3, 4, 6, 7]. GPs were asked to assess the challenges they perceive regarding these medical conditions and traits on a 10-point Likert scale. They could choose between ‘1 = not challenging at all’ and ‘10 = very challenging’. A high mean score indicates high challenge for the specific medical condition and the specific traits. Cronbach’s α of the measurement of different medical conditions was 0.858 and of different traits of patients was 0.739. The questionnaire of this survey was added as Additional file 1.

As an alternative for non-participation, a short-response-sheet was offered which evaluated only sociodemographic data like age and gender and reasons for non-participation.

### Data analysis

Analyses were performed using SPSS 24.0 (SPSS Inc., IBM). Continuous data was summarized using means and standard deviations. Categorical data was presented as frequency counts and percentage. Moreover, means, standard deviations and 95% confidence intervals of job satisfaction scale, challenging medical conditions and challenging traits of patients were reported. Pearson’s correlation was used to find out which the independent variables individual characteristics, aspect of job satisfaction, challenging medical conditions and challenging traits of patients showed a significant correlation with the dependent variable ‘overall job satisfaction’. Afterwards, a linear regression analyses were used to explore potential associations between the dependent variable ‘overall job satisfaction’ and independent variables which correlated significantly with the dependent variable. Additionally, the possibility for multicollinearity was considered. The variance inflation factor (VIF) and the value of tolerance were reported for the last step of both regression models. Values for VIF should not be over 5.0 and for tolerance not lower than 0.25 [14]. An alpha level of *P* < 0.05 was used for tests of statistical significance.

### Ethical approval

Ethical approval for this research study was obtained from the University of Luebeck in May 2015 (Approval No. 15–110). No additional data were evaluated.

## Results

**Fig. 1:**
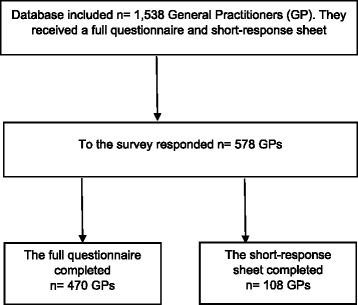
Flowchart of the study sample

Total response was 578 (38%). Out of these 470 returned the questionnaire (31%) and 108 returned the short-response-sheet (7%). A flowchart of the study sample is presented in Fig. 1. The demographic data of those participants returning the questionnaire and the short-response-sheet is shown in Table 1. These differed significantly concerning age and gender. The mean age of the participants returning the questionnaire was 55.0 (SD = 7.9) and of the short-response-sheet was 58.0 (SD 9.0). Over 34% of participants were women, whereas of the short-response-sheet 50% were women. Most physicians were practicing for more than 20 years (43%) or between 11 and 20 years (29%). Young professionals, defined as practicing for less than 5 years, were the smallest group (7%). More than half of the participants were located in medium-size towns. The others were located in urban (25%) or rural (18%) areas. GPs perceived 16% of their patients as challenging. Younger GPs perceived a higher rate of patients as challenging.

**Table 1:** Sociodemographic data of participants and short-response-sheet -participants (*n* = 577)

Characteristics^a^		Participants (*n* = 470)	short-response-sheet -participants (*n* = 107)	*p*-value
Age, mean (SD)		54.5 (7.9)	57.8 (9.0)	< 0.001
Gender, n (%)	Female	160 (34.0%)	55 (51.4%)	< 0.001
	Male	307 (65.3%)	51 (47.7%)	
Employment as GP, n (%)	< 5 years	35 (7.4%)	–	–
	5–10 years	95 (20.2%)	–	–
	11–20 years	134 (28.5%)	–	–
	> 20 years	203 (43.2%)	–	–
Location of GP’s practice, n (%)	Urban	119 (25.3%)	–	–
	Rural	86 (18.3%)	–	–
	Medium-size town	253 (53.8%)	–	–
Challenging patients, proportion (range)		15.96% (0–80%)	–	–

^a^n various due to missing data; *SD* standard deviation, *GP* general practitioner

Most of the short-response-sheet -participants (*n* = 53, 49.0%) claimed that they do not work as GPs but in a different specialty or already retired. Of the others 22% (*n* = 24) felt, that they had no time to complete the questionnaire. Another 14% (*n* = 15) stated that they do not participate in surveys in general.

Table 2 shows the mean score of the different items measuring job satisfaction. GPs were mostly satisfied with ‘colleagues and fellow workers’ (mean = 5.80). They were mostly dissatisfied with their ‘hours of work’ (mean = 4.20), ‘income’ (mean = 4.47) and ‘physical working condition’ (mean = 4.91).

**Table 2:** Descriptive statistics of job satisfaction (*n* = 470)

Aspects of job satisfaction^a^	Mean (SD)	CI 95%
Physical working condition	4.91 (1.55)	4.77–5.06
Freedom of working method	5.17 (1.46)	5.03–5.30
Colleagues and fellow workers	5.80 (1.26)	5.68–5.92
Recognition for work	5.44 (1.40)	5.31–5.57
Amount of responsibility	5.04 (1.58)	4.89–5.19
Income	4.47 (1.67)	4.31–4.62
Opportunity to use abilities	5.27 (1.43)	5.14–5.41
Hours of work	4.20 (1.82)	4.03–4.37
Amount of variety in job	5.49 (1.39)	5.36–5.62
Overall job satisfaction	5.41 (1.26)	5.29–5.53

^a^range from 1 “extreme dissatisfaction” to 7 “extreme satisfaction”; *SD* standard deviation, *CI* confidence interval

Table 3 shows the mean score of different medical conditions which were perceived as challenging. Somatization disorder, chronic pain and addiction to alcohol were ranked top three of challenging conditions with a mean score of more than seven points. Diabetes type 2, failure and high blood pressure had the lowest mean score on the scale (below 5 points).

**Table 3:** Descriptive statistics of challenging medical conditions of patients from the perspective of GPs

Medical Conditions^a^	Mean (SD)	CI 95%
Somatization disorder	7.42 (2.38)	7.20–7.65
Chronic pain	7.22 (2.34)	7.00–7.44
Addiction to alcohol	7.07 (2.45)	6.84–7.31
Schizophrenia	6.83 (2.56)	6.59–7.08
Depression	6.67 (2.38)	6.45–6.90
Anxiety disorder	6.57 (2.50)	6.34–6.81
Dementia	6.32 (2.56)	6.08–6.56
Obesity	5.90 (2.54)	5.66–6.14
Polypharmacy	5.68 (2.51)	5.44–5.92
Multimorbidity	5.22 (2.50)	4.98–5.45
Chronic renal failure	5.05 (2.48)	4.82–5.28
Diabetes type 2	4.87 (2.50)	4.64–5.11
Heart failure	4.03 (2.43)	3.80–4.26
High blood pressure	3.22 (2.14)	3.02–3.43

^a^range from 1 “not challenging at all” to 10 “very challenging”; *SD* standard deviation, *CI* confidence interval, *GP* general practitioner

Different challenging patient traits are presented in Table 4. Patients who experienced as aggressive and demanding were ranked high as challenging with a mean of 7.88 respectively 7.85. In contrast patients who experienced as critical and anxious were ranked low as challenging with a mean of 4.87 respectively 4.37.

**Table 4:** Descriptive statistics of challenging traits of patients from the perspective of GPs

Characteristic of patients^a^	Mean (SD)	CI 95%
Aggressive	7.88 (2.56)	7.64–8.12
Demanding	7.85 (2.27)	7.64–8.06
Unfriendly	6.77 (2.72)	6.52–7.02
Limited compliance	6.24 (2.64)	6.00–6.48
Obsessive-compulsive personality	6.00 (2.34)	5.78–6.22
Person with a lot of questions	5.68 (2.53)	5.44–5.91
Critical	4.87 (2.41)	4.65–5.09
Anxious	4.37 (2.75)	4.11–4.62

^a^range from 1 “not challenging at all” to 10 “very challenging”; *SD* standard deviation, *CI* confidence interval, *GP* general practitioner

The results of the correlation showed that the demographic data ‘age’, ‘gender’ and ‘employment as GP’ correlated strong with the dependent variable “overall job satisfaction”. The different aspects of job satisfaction showed a strong correlation to the dependent variable.

For the medical conditions ‘heart failure’, ‘diabetes type 2’, ‘chronic renal failure’, and ‘anxiety disorder’ and for the patient traits ‘anxious’, ‘unfriendly’, ‘obsessive-compulsive personality’, and ‘person with a lot of questions’ a strong correlation to the dependent variable ‘overall job satisfaction’ was found.

Table 5 shows the linear regression analysis of the individual characteristics, aspects of job satisfaction, challenging medical conditions and traits of patients which correlated significantly with the outcome variable ‘overall job satisfaction’. A model with an explained variance with more than 71% (R^2^~ 0.714) on the outcome variable ‘overall job satisfaction’ was carried out. The higher overall satisfaction was associated with lower years of employment as a GP and higher satisfaction concerning nearly all aspect of job satisfaction except for recognition for work and income. The statistics of collinearity ranged between 2.406 (VIF-value), 0.416 (tolerance value) for ‘employment as GP’ and 1.062 (VIF-value), 0.941 (tolerance value) for ‘amount of variety in job’.

**Table 5:** Associations of individual characteristics, different aspects of job satisfaction and challenging medical condition and characteristics of patients on the outcome variable overall job satisfaction (results of linear regression analysis, under specification of standardized beta coefficient, α = 5%)

	Variables	β	*p*-value
Characteristic of participants	Age	0.097	0.023
	Gender	0.046	0.128
	Employment as GP	−0.091	0.033
Aspects of job satisfaction	Physical working condition	0.367	< 0.001
	Freedom of working method	0.096	0.003
	Colleagues and fellow workers	0.078	0.007
	Recognition for work	0.051	0.078
	Amount of responsibility	0.240	< 0.001
	Income	0.052	0.072
	Opportunity to use abilities	0.244	< 0.001
	Hours of work	0.108	0.003
	Amount of variety in job	0.061	0.033
Challenging medical conditions of patients	Heart failure	0.017	0.675
	Diabetes type 2	−0.032	0.399
	Chronic renal failure	0.034	0.383
	Anxiety disorder	−0.035	0.305
Challenging traits of patients	Anxious	−0.008	0.818
	Unfriendly	−0.010	0.751
	Obsessive-compulsive personality	−0.031	0.343
	Person with a lot of questions	−0.034	0.278
R^2^		0.714	

*GP* general practitioner

## Discussion

To our knowledge there has been little research on difficult medical encounters especially for their association with job satisfaction on primary care physicians in Germany. Our sample of participating GPs are comparable to the whole sample of GPs in Germany concerning age but differs slightly by gender, 34.0% women in our sample comparing to 43.9% in the whole sample of GPs in Germany [15]. Moreover, the results showed that our participants were mostly satisfied with their colleagues but dissatisfied with their income and working hours which is comparable to other studies with GPs not only in Germany [16–18].

Furthermore, our results confirms findings from previous international studies dealing with challenging encounters in primary care. It could be shown that GPs perceive 16% of their patients as challenging [1, 2]. In accordance with the statement to complex individual humans it can be assumed that both - GP and patient - meeting in a specific situation should initiated an acceptable work alliance [19]. However, for this specific situation it is a challenge, different medical conditions and traits of patients will be perceived as challenging and there is a clash of two complex systems, the system from the GP and the system from the patient. The encounter between GP and patient could be experienced as challenging from the perspective of GP and could be increased if emotional or behavioural factors from patients overlap or influence the consultation process. Moreover, this specific situation could lead to the perception a difficult encounter.

Especially ‘somatization disorder’, ‘chronic pain’ and ‘addiction to alcohol’ were perceived as challenging medical conditions by the participating GPs. The chronic conditions like ‘high blood pressure’, ‘heart failure’ and ‘diabetes type 2’ showed the lowest rate of challenging medical conditions. Consistently as challenging perceived are psychiatric diseases, whereas diseases of the cardiovascular system are considered least challenging [4, 6]. It has been observed that ‘aggressive patients’ have the highest rate within the challenging traits of patients which is comparable to an interview study with family physicians. This study identified aggressive patients as the most difficult ones [20]. It can be assumed that the careful handling of challenging patients especially with psychiatric diseases could be an important part for the training to get a GP. The German training regulation for GPs (‘Weiterbildungsverordnung für Ärzte’) may be a cause for the challenges GPs come up with when treating patients with mental disorders, since it presumably does not stipulate the acquisition of required skills in the diagnosis and treatment of psychiatric disorders, like depression. This should have high priority due to high prevalence and therefore relevance. About 1 % of German postgraduates do psychiatry rotations, thus structured training is rare [21]. In Germany, the lifetime prevalence of diagnosed depression is 15% in women and 8% in men and most of them are seen and managed in primary care [22]. In addition, depression is a costly disease not so much due to therapy costs but due to missed workdays [23].

The rotation curricula for the training of GPs in other countries, where GPs perceive psychiatric disorders as challenging as well, do not contain the acquisition of broad skills in psychiatry, neither [24]. This might explain the scientific discourse concerning the quality of care when it comes to treating patients with depression in primary care settings [25]. Thus future studies should address the questions, in what amount psychiatric competences are gained during the postgraduate training in general practises and if GPs with a mandatory rotation in psychiatry perceive these conditions as less challenging.

Surprisingly, challenging encounters described with medical conditions and traits of patients are not associated with overall job satisfaction of participating physicians. However, motivational aspects at work expressed in terms of different intrinsic and extrinsic factors are strongly associated with overall job satisfaction. It can be assumed that the feeling at the job is more relevant and generalizable than the challenging encounters for our sample. However, our results are in contrast to a study which found an interaction between lower job satisfaction and higher reporting challenging encounters [7].

### Strength and limitations

This was the first cross-sectional study performed with all GPs of a complete federal state in Germany addressing challenging encounters. The study benefited from the usage of a well-known instrument which was validated in a large cohort of Australian medical practitioner [13]. The job satisfaction scale was already used in different studies about job satisfaction in primary care in Germany [16–18]. The job satisfaction scale was not specific validated for our sample. The response rate was 38% relatively high in contrast to the statement by Kelley et al. [26]. They assumed for postal questionnaire surveys a response rate of 20% as normal for such surveys [26]. However, there is a risk of selection bias of highly motivated GPs in our results who answered to the questionnaire. Interestingly, short-response-sheet responders in our study were slightly older and more often female comparing to responders. This corresponds with findings of earlier studies that female GPs are somewhat less willing to participate in studies in primary care [27]. In addition, a potential lack of accuracy and completeness of the GP’s remembrance of patients and situations might have led to recall bias and therefore a systematic error. Furthermore, the list of challenging medical conditions and traits included a high proportion of different psychiatric diseases which could influence GP’s response behaviour. In addition, this was an exploratory study; *p* values should be interpreted carefully. Moreover, there are no clear statements in the literature concerning the statistical analysis of surveys using Likert scales [28, 29]. Therefore, we handled the Likert scales as an interval which could implicated a potential statistical bias. Finally, this was a cross-sectional study, and thus, we must be cautious to derive causal links from these findings.

## Conclusions

German GPs perceive 16% of their patients as challenging. Psychiatric disorders such as somatization disorders and chronic pain are perceived as challenging. Moreover, the data shows that aggressive and demanding patients influence the perception as challenging encounter. It can be assumed that to deal with these attributes a specific training of communication skills during postgraduate training but also for experienced GPs would be necessary. The investigation in a Continuing Professional Development including communication training, conflict management and dealing with teams might be a supporting function for working as a GP and to ensure a good quality of care. Interestingly this study indicates that challenging encounter in terms of challenging medical conditions and traits of patients do not affect GP’s job satisfaction. However, more research is needed concerning the connection between the perception of the complexity of challenging encounter and their impact on quality of care in longitudinal studies.

## Additional file

Additional file 1: Questionnaire “Difficult medical encounters and job satisfaction”. The questionnaire for the presented survey is available as additional file. (PDF 28 kb)

## Abbreviations

BBSR: Federal Institute for Research on Building, Urban Affairs and Spatial Development
CI: Confidence interval
GP: General practitioner
SD: Standard deviation
SPSS: Statistical package of social science
STROBE-Guidelines: Strengthening the Reporting of Observational Studies in Epidemiology
VIF: variance inflation factor
