# Supplementary material for: Difficult medical encounters and job satisfaction - results of a cross sectional study with general practitioners in Germany
Source: BMC Fam Pract. 2018 May 9;19:57. doi: 10.1186/s12875-018-0747-0 (PMC5944023; doi:10.1186/s12875-018-0747-0)
Supplement: Supplementary file 1 — Questionnaire “Difficult medical encounters and job satisfaction”. The questionnaire for the presented survey is available as additional file. (PDF 28 kb) [file 12875_2018_747_MOESM1_ESM.pdf]

UNIVERSITÄT ZU LÜBECK

Questionnaire: Difficult medical encounters and job satisfaction

Dear colleagues,

Thank you for your participation. This survey is anonymous. Your answers will be analyzed from the Institute of Family Medicine, University Hospital Campus Luebeck. The information provided will be treated as strictly confidential. These data will not be released to third parties.

1. How satisfied are you with your…?

	**extreme dissatisfied**						**extreme satisfied**
	**1**	**2**	**3**	**4**	**5**	**6**	**7**
**a.** Physical working condition	☐	☐	☐	☐	☐	☐	☐
**b.** Freedom of working method	☐	☐	☐	☐	☐	☐	☐
**c.** Colleagues and fellow workers	☐	☐	☐	☐	☐	☐	☐
**d.** Recognition for your work	☐	☐	☐	☐	☐	☐	☐
**e.** Amount of responsibility	☐	☐	☐	☐	☐	☐	☐
**f.** Income	☐	☐	☐	☐	☐	☐	☐
**g.** Opportunity to use your abilities	☐	☐	☐	☐	☐	☐	☐
**h.** Hours of work	☐	☐	☐	☐	☐	☐	☐
**i.** Amount of variety in your job	☐	☐	☐	☐	☐	☐	☐
**j.** Overall job satisfaction	☐	☐	☐	☐	☐	☐	☐

UNIVERSITÄT ZU LÜBECK

Challenging encounters

2. **Please estimate: How many patients are challenging in your practice?** |_|_|_| %

3. **Please estimate: Which traits of patients are challenging for your:**

Not at all	1	2	3	4	5	6	7	8	9	10	Very
**a.** Anxious	☐	☐	☐	☐	☐	☐	☐	☐	☐	☐	
**b.** Unfriendly	☐	☐	☐	☐	☐	☐	☐	☐	☐	☐	
**c.** Demanding	☐	☐	☐	☐	☐	☐	☐	☐	☐	☐	
**d.** Aggressive	☐	☐	☐	☐	☐	☐	☐	☐	☐	☐	
**e.** Obsessive-complusive personality	☐	☐	☐	☐	☐	☐	☐	☐	☐	☐	
**f.** Critical	☐	☐	☐	☐	☐	☐	☐	☐	☐	☐	
**g.** Person with a lot of question	☐	☐	☐	☐	☐	☐	☐	☐	☐	☐	
**h.** Limited compliance	☐	☐	☐	☐	☐	☐	☐	☐	☐	☐	

4. **Please estimate: Which condition are challenging for you in your care of patients:**

Not at all	1	2	3	4	5	6	7	8	9	10	Very
**a.** High blood pressure	☐	☐	☐	☐	☐	☐	☐	☐	☐	☐	
**b.** Heart failure	☐	☐	☐	☐	☐	☐	☐	☐	☐	☐	
**c.** Obesity	☐	☐	☐	☐	☐	☐	☐	☐	☐	☐	
**d.** Diabetes type 2	☐	☐	☐	☐	☐	☐	☐	☐	☐	☐	
**e.** Chronic renal failure	☐	☐	☐	☐	☐	☐	☐	☐	☐	☐	
**f.** Chronic pain	☐	☐	☐	☐	☐	☐	☐	☐	☐	☐	
**g.** Dementia	☐	☐	☐	☐	☐	☐	☐	☐	☐	☐	
**h.** Addiction to alcohol	☐	☐	☐	☐	☐	☐	☐	☐	☐	☐	
**i.** Depression	☐	☐	☐	☐	☐	☐	☐	☐	☐	☐	
**j.** Somatization disorder	☐	☐	☐	☐	☐	☐	☐	☐	☐	☐	
**k.** Anxiety disorder	☐	☐	☐	☐	☐	☐	☐	☐	☐	☐	
**l.** Schizophrenia	☐	☐	☐	☐	☐	☐	☐	☐	☐	☐	
**m**. Multimorbidity	☐	☐	☐	☐	☐	☐	☐	☐	☐	☐	
**n.** Polypharmacy	☐	☐	☐	☐	☐	☐	☐	☐	☐	☐	

UNIVERSITÄT ZU LÜBECK

Demograhic and practice characteristics

5. **How old are you?** ☐☐ years

6. **You are …** ☐ male ☐ female

7. **How many years did you working as a GP?** ☐ < 5 ☐ 5-10 ☐ 11-20 ☐ > 20 years

8. **In which district did you working?** *(Please indicate your location designation.)* ☐☐☐

9. **What kind of aspects did you missing in the survey?**

____

____

____

____

____

Thank you for your participation!
